# Central Retinal Sensitivity Decline in *RPGR*-Related Retinal Phenotypes

**DOI:** 10.1016/j.ajo.2025.11.002

**Published:** 2026-02

**Authors:** Amandeep Singh Josan, Laura J. Taylor, Shabnam Raji, Jasmina Cehajic-Kapetanovic, Robert E. MacLaren

**Affiliations:** From the Nuffield Laboratory of Ophthalmology, Department of Clinical Neurosciences, University of Oxford; and Oxford Eye Hospital, Oxford University Hospitals NHS Foundation Trust, Oxford, United Kingdom

## Abstract

**Purpose:**

To characterize the natural history of central retinal sensitivity decline in *RPGR*-associated retinopathy and to evaluate and quantify phenotypic differences in disease progression using microperimetry-derived metrics.

**Design:**

A retrospective cohort study.

**Subjects:**

50 patients with genetically confirmed *RPGR* mutations (36 rod-cone, 14 cone-rod dystrophy [CORD], contributing 357 microperimetry assessments) were recruited.

**Methods:**

Central retinal function was assessed using the standard 68-point MAIA microperimeter grid pattern after 20 minutes of dark adaptation and without dilation. Mean sensitivity across the central 4 (MS4) and 16 (MS16) loci were extracted and analyzed post hoc. Longitudinal decline rates were modeled using generalized linear mixed models, and survival analyses were conducted using Cox proportional hazards models with random intercepts and robust clustering to account for repeated measures and intra-subject correlation.

**Main outcome measures:**

The rate of retinal sensitivity decline was measured.

**Results:**

CORD patients exhibited significantly faster rates of central retinal sensitivity decline. The annual MS16 decline rate was 10.8% (95% CI: 6.6, 14.8) in CORD vs 5.1% (95% CI: 2.9, 7.1) in rod-cone patients (*P* = .02). MS4 declined by 14.9% (95% CI: 10.2, 19.4) annually in CORD compared to 4.1% (95% CI: 1.2, 6.8) in rod-cone (*P* < .001). The median survival age to total loss of sensitivity for MS16 was 25.1 years in CORD and 33.1 years in rod-cone (*P* = .01), and for MS4, 27.2 years and 33.3 years, respectively (*P* = .02). Hazard ratios demonstrated an 8.2-fold (MS16) and 7.2-fold (MS4) increased instantaneous risk of central vision loss in CORD compared with rod-cone patients.

**Conclusions:**

This study reveals significantly different rates of functional decline between *RPGR* phenotypes, with CORD patients at markedly higher risk of early central vision loss. These findings support the use of microperimetry as a sensitive outcome measure and provide critical survival data for clinical trial design, patient counseling, and therapeutic prioritization. Patients with cone-dominated phenotypes may benefit from earlier intervention.

Retinitis pigmentosa (RP) encompasses a broad spectrum of genetically heterogeneous disorders characterized by progressive retinal degeneration primarily affecting rod and cone photoreceptors.^1^ The disease typically manifests with night blindness and peripheral visual field loss, eventually progressing to severe central vision impairment or complete blindness.^2^^,^^3^ X-linked RP, primarily caused by mutations in the Retinitis Pigmentosa GTPase Regulator (*RPGR*) gene, represents one of the most severe and common forms.

*RPGR* encodes a protein localized primarily to the photoreceptor connecting cilium, where it plays a critical role in protein trafficking between the inner and outer segments of photoreceptors.^4^^,^^5^ The gene contains 2 major isoforms: a constitutive isoform encoded by exons 1-19 and a retina-specific alternatively spliced isoform containing a unique terminal exon, ORF15, which is a mutational hotspot accounting for approximately 60% of all disease-causing *RPGR* mutations.^6^^,^^7^ The ORF15 region contains a highly repetitive purine-rich sequence that predisposes it to frameshift mutations, which have been associated with variable phenotypic presentations.^8^^,^^9^ Mutations within the *RPGR* gene lead to significant variability in clinical presentations, ranging from predominantly rod-cone dystrophies to aggressive cone-rod dystrophies (CORD).^10^

Rod-cone dystrophy typically initiates with peripheral vision loss, gradually progressing centrally over many decades, in keeping with traditional RP pathophysiology understanding and accounting for 70% of *RPGR*-associated retinopathy cases. CORD, however, is characterized by a more rapid central involvement with marked vision deterioration, profoundly affecting visual acuity and quality of life in relatively short periods. This form occurs in approximately 25% of *RPGR*-associated retinopathy cases.^8^^,^^11^

This known phenotypic heterogeneity poses significant challenges for clinicians in providing accurate prognostic information and for researchers designing appropriate clinical trials with meaningful end points that can capture disease progression accurately. The maintenance of central and paracentral vision is paramount for most patients' quality of life and independence, with central retinal sensitivity representing a critical functional parameter that correlates strongly with everyday visual tasks.^12^, ^13^, ^14^ Microperimetry has emerged as a valuable tool for quantifying macular retinal function through precise threshold sensitivity measurements correlated with specific retinal locations.^15^^,^^16^ Unlike conventional perimetry, microperimetry incorporates fundus tracking technology, enabling precise spatial correlation between anatomical features and functional sensitivity, making it particularly valuable for monitoring macular function in progressive retinal conditions.^17^

Despite advancements in genetic diagnostics and increasing interest in *RPGR* as a target for gene therapy,^18^^,^^19^ comprehensive longitudinal studies specifically quantifying the rate of visual function decline in *RPGR*-associated retinopathy using standardized functional end points such as microperimetry remain scarce.^20^ Such data are essential not only for improving prognostic accuracy and patient counseling but also for designing appropriate clinical trials with meaningful end points that can capture disease progression accurately, particularly when designing treatments that may have variation in efficacy when delineating disease by phenotype. Although differences in progression rates are expected because of the phenotypic classification being based on many of these very attributes, precise quantification of rates of decline using microperimetry have not been extensively explored before.

The present study addresses this knowledge gap by using microperimetry to systematically analyze the natural history of central retinal sensitivity decline in patients with genetically confirmed *RPGR* mutations. Through detailed survival analysis and Cox regression modeling, we aim to characterize the trajectory and rate of disease progression across different phenotypic presentations as well as establish reference data that can inform both clinical management and future therapeutic trials targeting this severe form of inherited retinal degeneration.

## METHODS

This retrospective cohort natural history analysis included data from patients diagnosed with *RPGR*-related retinopathy across longitudinal and cross-sectional assessments performed at specialized retinal centers. The study adhered to the tenets of the Declaration of Helsinki, and written informed consent was obtained from all participants.

Patients were stratified into 2 phenotypic groups—rod-cone dystrophy (rod-cone) and cone-rod dystrophy (CORD)—based on clinical presentation and retinal imaging characteristics. Phenotypic classification was undertaken by a clinical expert (J.C.K.) using multimodal imaging, electrophysiology, and retinal sensitivity using microperimetry. Detailed methodology with examples are described in previous publication.^8^ Patients with more aggressive phenotypes characterized as cone/cone-rod or cone-only dystrophy (COD) were excluded from this analysis to highlight and quantify differences between the more common variations between rod-cone vs CORD phenotypic presentations. Microperimetry decline in cone/cone-rod and COD tend to be severe, with obvious differences from that of rod-cone and CORD, but the rare presentations of these more severe phenotypes make quantitative comparisons more challenging.

Quantitative assessment of retinal function was conducted using the MAIA microperimeter (CenterVue). The standardized testing protocol employed a 4-2 threshold strategy with a Goldmann III stimulus size, presented for 200 ms against a background luminance of 1.27 cd/m². A standard 68-point grid pattern was used, covering the central 10° of the retina with a consistent 2° spacing between stimulus loci. Two key metrics were then extracted and considered for the survival analysis, specifically evaluating central retinal sensitivity through mean sensitivity values of (1) the central 4 loci (MS4) and (2) the central 16 loci (MS16), with both metrics expressed in decibels (dB). Tests with poor reliability (fixation losses >30%) were excluded from the analysis as per standard protocol. Those patients with a cone-only or predominantly cone phenotype were also excluded. In addition, there was no formal learning tests undertaken.

Survival analysis or time-to-event analysis is typically composed of data comprising of multiple forms, which were reviewed by McGuinness and associates.^21^ Here we summarize them as follows:

1. Left censoring: Complete MS16/MS4 loss occurred before the first visit but patient survival data are still included (ie, zero values included in analysis).
2. Right censoring: Complete MS16/MS4 loss did not occur even by the last visit but patient survival data are still included.
3. Interval censoring: Complete MS16/MS4 loss occurs between visits but exact time is not known. Patient survival data are still included.
4. Left truncation: Patient data are entirely excluded from analysis if complete MS16/MS4 loss occurred before their first observation, that is, no nonzero recorded MS16/MS4 values. Exclusion introduces potential biases against patients with aggressive forms of disease.
5. Right truncation: Patient data are entirely excluded from analysis if complete MS16/MS4 loss did not occur before the last observation, that is, no zero recorded MS16/MS4 values. Exclusion introduces potential biases against patients with mild forms of disease.

In this study, all available data are included and there is no left or right truncation. Being a retrospective study and because of the progressive nature of inherited retinal diseases, left, right, and interval censoring is extremely common and forms the basis of our data analysis here.

### STATISTICAL ANALYSIS

All statistical analyses were performed using R software, version 4.1.0,^22^ with a significance threshold set at *P* <.05. Descriptive statistics were collected for demographic, clinical, and microperimetry parameters. For longitudinal analysis of retinal sensitivity decline, generalized linear mixed models (GLMMs) using the lme4 package^23^ were used to account for within-subject correlation from repeated measurements. The models incorporated patient age and phenotype (rod-cone dystrophy vs CORD) as fixed effects with interactions, and with patient ID included as a random intercept and random slope model. Separate models were developed for both MS4 and MS16 outcomes to comprehensively characterize central retinal function decline, with both metrics being of particular relevance.

Survival analyses were conducted using the *survival* package^24^ and mixed effects Cox proportional hazards regression models constructed using the *coxme* package^25^ to characterize the time course of functional vision loss for MS4 and MS16 taking repeated measures and nesting of both eyes into account. The *coxph* function^24^ with a cluster term to account for 2 eyes nested within each patient and each patient tested repeatedly was used to generate survival curves and estimate median survival ages, or the median age where there is a 50% probability of retinal sensitivity being extinguished. The cluster term uses robust variance analysis (clustered Huber-White sandwich method) to estimate variances rather than a full random intercepts model as used in the *coxme* package. Thus, the method of generation of 95% CIs differs for the median ages when compared to the hazard ratios (HRs) because of this slight difference in variance estimations.

In the context of this study, the hazard rates are the instantaneous risk, at any given time, that complete MS16/MS4 loss occurs. HRs are therefore the ratio of the instantaneous risk of an event occurring in one group vs the other group. For example, in MS16, an HR >1 would be interpreted as a greater risk of complete MS16 loss in CORD compared with rod-cone patients at any given moment. HRs are often confused with cumulative measures of risk such as risk ratios. Risk ratios assess the cumulative risk of MS16 loss in one group compared with the other at the *end* of the study and is a measure of the overall risk. HRs, in contrast, are an *instantaneous* measure of risk at any given moment. Risk ratios, therefore, are more relevant in interventional trials where an improvement at the end of the study is sought.

## RESULTS

### DEMOGRAPHIC AND CLINICAL CHARACTERISTICS

**FIGURE 1:**
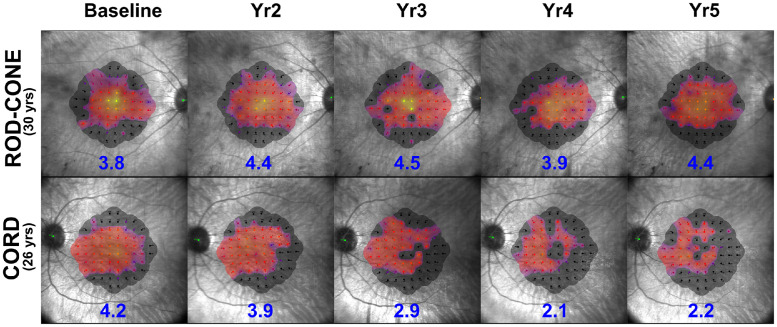
Microperimetry progression plots of a typical rod-cone patient (top) and CORD patient (bottom) with mean sensitivity (decibels) as an average of all points highlighted in blue. *RPGR*-associated rod-cone patients characteristically retain relatively good central retinal sensitivity levels exhibiting a fairly uniform decline as eccentricity increases. CORD patients, on the other hand, tend to demonstrate early central foveal involvement affecting the central 4 microperimetry points. Later stage rod-cone patients can appear like earlier-stage CORD patients (see rod-cone year 5 compared with baseline CORD plots). In such cases, other modalities, such as OCT, color fundus imaging, fundus autofluorescence, and electroretinography, can help to distinguish between phenotypes. CORD = cone-rod dystrophy, OCT = ocular coherence tomography.

A total of 50 male patients (36 of rod-cone and 14 of CORD phenotypes) with genetically confirmed *RPGR* mutations were included in the study, totaling 357 microperimetry assessments. Example microperimetry changes for each phenotpe are shown in Figure 1.

Relevant clinical characteristics of the cohort are summarized in the Table. The mean age at baseline evaluation was 31.3 (14) years for the rod-cone phenotype and 25.4 (11.7) years for the CORD phenotype (difference *P* = .14). Of those patients who were examined on more than 1 occasion (35 of 50 patients), the mean follow-up duration for all patients was 4.4 (2.3) years since first examination. Split by phenotype, the mean follow-up duration was 4.3 (2.3) years for rod-cone and 4.5 (2.5) years for CORD phenotypes (difference *P* = .92). Mean MS16 at baseline was 11.1 (8.2) dB for the rod-cone phenotype and 6.8 (5.8) dB for the CORD phenotype (difference *P* = .04). Mean MS4 at baseline was 14.3 (8.2) dB for the rod-cone phenotype and 8.9 (6.8) dB for the CORD phenotype (difference *P* = .02).

**TABLE:** Descriptive statistics for patients included across CORD and rod-cone phenotypes.

Phenotype	Number of Patients	Number of Examinations	Age, y, Mean (SD)	MS16, Mean (SD), dB	MS4, Mean (SD), dB
CORD	14	102	25.4 (11.7)	6.8 (5.8)	8.9 (6.8)
Rod-cone	36	255	31.3 (14)	11.1 (8.2)	14.3 (8.2)

CORD = cone-rod dystrophy, MS16 = mean sensitivity across the central 16 points that is most relevant for clinical trials, MS4 = mean sensitivity of the central 4 points that is most relevant for visual acuity and potential visual impairment certification.

### MEAN SENSITIVITY OF THE CENTRAL 16 POINTS (MS16)

**FIGURE 2:**
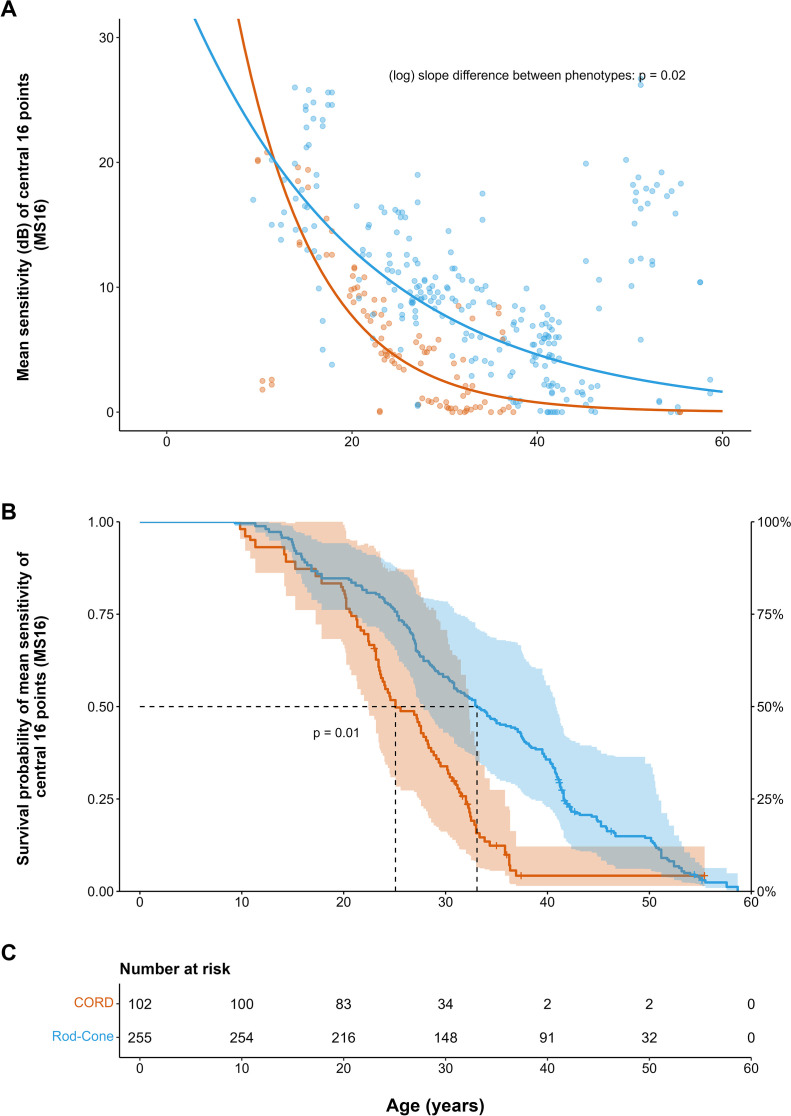
Linear mixed model regression and Cox proportional hazard survival plot for MS16. MS16 = mean sensitivity across the central 16 points that is most relevant for clinical trials.

Retinal sensitivity decline rates calculated from generalized linear mixed modeling demonstrated that rod-cone dystrophy patients exhibited a slower decline, with a half-life of the rate of decline in the central 16 points (MS16) of 13.3 (95% CI: 9.4, 23.2) years compared with 6.0 (95% CI: 4.3, 10.1) years in CORD patients (difference *P* = .02) (Figure 2, A). This equates to a 5.1% (95% CI: 2.9, 7.1) and 10.8% (95% CI: 6.6, 14.8) annual reduction in MS16 for rod-cone and CORD patients, respectively.

Mixed effects Cox survival analysis revealed marked differences in disease progression between rod-cone and CORD phenotypes. The median survival age for the mean sensitivity of the central 16 points (MS16) was 33.1 (95% CI: 27.8, 40.8) years for rod-cone and 25.1 (95% CI: 22.4, 32.3) years for CORD patients (difference *P* = .01) (Figure 2, B). HR for complete loss of MS16 was 8.2 (95% CI: 1.8, 36.9), which can be interpreted as follows: at any given time, there is an 8.2 times increased risk of MS16 extinction in CORD compared with rod-cone phenotype patients (*P* = .01).

### MEAN SENSITIVITY OF THE CENTRAL 4 POINTS (MS4)

**FIGURE 3:**
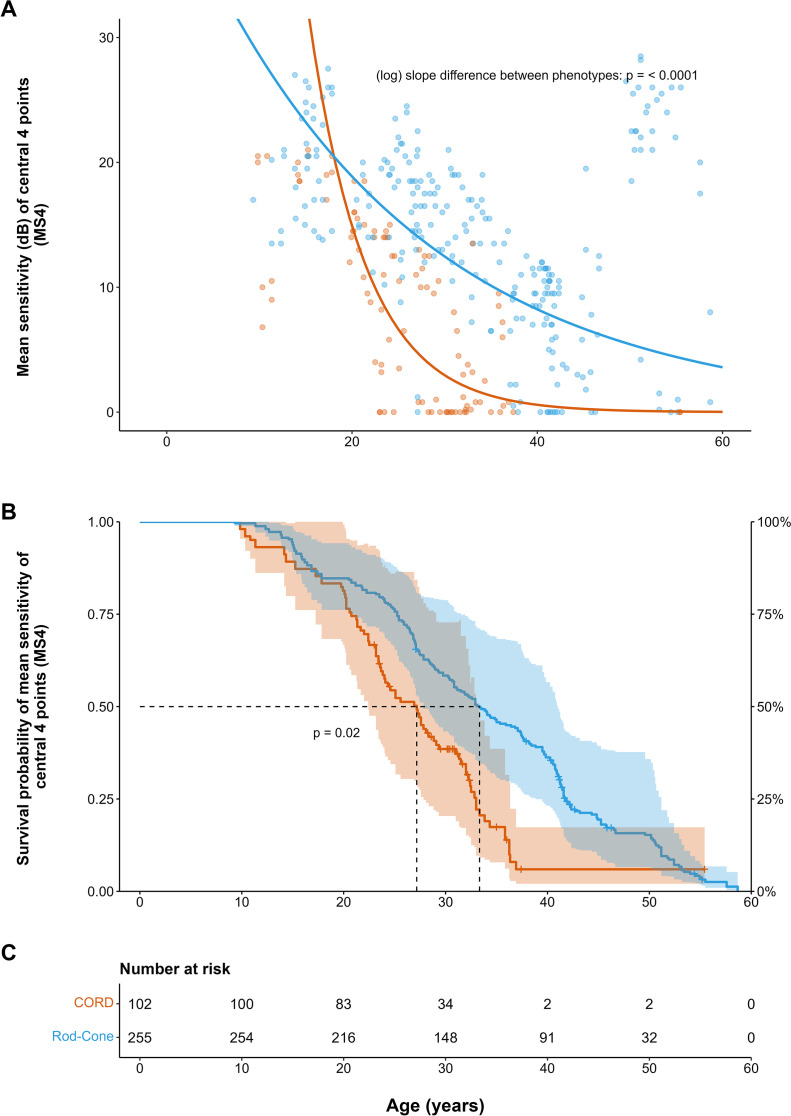
Linear mixed model regression and Cox proportional hazard survival plot for MS4. MS4 = mean sensitivity of the central 4 points that is most relevant for visual acuity and potential visual impairment certification.

The half-life of the rate of decline in the mean sensitivity of the central 4 points (MS4) was 16.7 (95% CI: 9.8, 55.2) years in rod-cone dystrophy patients vs just 4.3 (95% CI: 3.2, 6.4) years in CORD patients (difference *P* < .001) (Figure 3, A), highlighting the differing severity and geographic presentations of the 2 phenotypes (Figure 1). This equates to a 4.1% (95% CI: 1.2, 6.8) and 14.9% (95% CI: 10.2, 19.4) annual reduction in MS4 for rod-cone and CORD patients, respectively.

The median survival age for the mean sensitivity of the central 4 points (MS4) was 33.3 (95% CI: 27.8, 41) years for rod-cone and 27.2 (95% CI: 22.4, 33.3) years for CORD patients (difference *P* = .02) (Figure 3, B). HR for complete loss of MS4 was 7.2 (95% CI: 1.3, 38.9), which, again, can be interpreted as follows: at any given time, there is a 7.2 times increased risk of MS4 extinction in CORD compared with rod-cone phenotype patients (*P* = .02).

Hence, Cox regression analyses reveal phenotypic presentation as a significant influencing factor in determining disease progression, with CORD patients demonstrating significantly faster decay of MS4 and MS16 as expected. These particular metrics are relevant measures that translate to how well patients perform central vision tasks, and as guidance for clinical trial enrollment and monitoring frequency.

Thus, the main findings of this analysis are as follows:

1. MS16 decreases by 5.1% (95% CI: 2.9, 7.1) and 10.8% (95% CI: 6.6, 14.8) annually for rod-cone and CORD patients, respectively.
2. MS4 decreases by 4.1% (95% CI: 1.2, 6.8) and 14.9% (95% CI: 10.2, 19.4) annually for rod-cone and CORD patients, respectively.
3. There is an 8.2 (95% CI: 1.8, 36.9) times increased instantaneous risk of complete MS16 loss in CORD compared with rod-cone phenotype patients.
4. There is a 7.2 (95% CI: 1.3, 38.9) times increased instantaneous risk of complete MS4 loss in CORD compared with rod-cone phenotype patients.

## DISCUSSION

The use of microperimetry provides highly sensitive and spatially precise functional data that correlates well with patients' visual experience and quality of life.^14^^,^^26^^,^^27^ The inclusion of both cross-sectional and longitudinal observations enhances the robustness of our progression estimates. Furthermore, the application of statistical modeling techniques, including mixed-effects models and survival analysis, appropriately accounts for the complex data structure and provides clinically interpretable metrics of disease progression. The annual reductions in MS16 highlights the potential window for recruitment onto clinical trials, because the central 16 microperimetry points are of relevant interest to regulatory authorities. However, the annual reduction rate of MS4 may be of greatest interest to the patient and genetic counselors who may wish to advise on the likely disease course and probable age at which their central foveal vision may be most affected.

The findings here compare favorably with a recent literature review.^28^ For example, the annual decline rates for MS16 of 5.1% for rod-cone and 10.8% for CORD shown above compare well with the range of 4.2% to 13.3% annual decline in static perimetry across all *RPGR* studies examined. These studies did not classify by phenotype.

The results of this comprehensive study highlight and quantify the significant variability in central retinal disease progression rates among patients with *RPGR*-associated retinopathy, clearly differentiating between rod-cone and CORD phenotypes. The rapid progression observed in the CORD phenotype, in particular, may justify the clinical need for earlier interventions once available and more frequent monitoring protocols.

The phenotype-specific progression data established in this study have several important clinical implications. First, they emphasize the necessity of early genetic diagnosis and phenotyping to identify patients with *RPGR* mutations, particularly those with variants associated with the CORD phenotype who face an imminent risk of severe central vision loss. For these rapidly progressing patients, more frequent monitoring may be warranted, and expedited consideration for emerging therapeutic interventions should be prioritized. Importantly, distal ORF15 variants associated with cone-dominated phenotypes are known to impair *RPGR* glutamylation due to disrupted interaction with TTLL5,^8^ supporting the use of full-length, codon-optimized *RPGR* constructs in gene therapy to restore normal protein function and improve therapeutic outcomes in this subgroup at a timely age.

Second, for rapidly progressing CORD patients, shorter trials with sensitive functional end points may be appropriate, whereas the more gradually progressing rod-cone dystrophy population may require longer observation periods or alternative outcome measures to detect therapeutic efficacy. In addition, the early identification of phenotype associated with more severe disease courses may inform the eligibility criteria for therapeutic interventions. As gene therapy approaches for *RPGR*-associated retinopathy advance through clinical trials, prioritizing patients with high-risk genetic variants may optimize the risk-benefit profile by targeting those with the greatest unmet need and therapeutic window opportunity.

The authors acknowledge several limitations in this analysis. First, the retrospective design introduces the potential for selection bias, as those patients with more severe or rapidly progressing disease are likely to attend hospital clinics. Second, phenotyping more accurately follows a spectrum of presentations rather than the simplified rod-cone vs CORD categorizations applied here; thus, within each phenotypic group, a significant degree of variation was evident. Among rod-cone dystrophy patients, some exhibited early microperimetry loss, whereas others demonstrated preserved sensitivity until much later in life. Notably, a cluster of rod-cone data points in FIGURE 1, FIGURE 2 around the age of 50 years, showing relatively preserved MS16 and MS4 values, corresponds to longitudinal measurements from 2 related individuals. Ongoing investigations aim to determine whether a distinct molecular or genetic mechanism underlies this unusually mild phenotype.

In addition, unknown precise ages of disease onset presents as a previously established confounding effect preventing generalizable statements on the expected absolute ages of retinal sensitivity extinction. However, the Cox regression performed in this study had demonstrated that once retinal disease initiates (ie, sometime prior to the first attendance at the eye hospital), retinal sensitivity follows a relatively predictable rate of decay for both rod-cone and CORD phenotypes regardless of the heterogenous age of onset.

Therefore, we place less emphasis on the median survival age of MS16 and MS4 findings (as these are highly subject dependent, being possibly due to significant differences in baseline statistics where estimates are likely biased toward more severe disease more often seen at the eye hospital) and a greater emphasis on the rates of decay and HRs of MS16 and MS4 loss between rod-cone and CORD phenotypes. This is akin to analysis and commonalities of regression slopes (which has low between subject variation) rather than regression intercepts (which suffers from a high degree of between subject variation). These measures provide accurate indicators of the differing microperimetry survival times between phenotypes.

Further, although this study focuses on survival of functional measures, future studies incorporating multimodal structure-function correlation and information regarding lifestyle factors and comorbidities would further enhance our understanding of the relationship between anatomical degeneration and functional decline in *RPGR*-related retinopathy.

These findings provide compelling evidence supporting genetic testing as a fundamental component of prognostic evaluation and individualized patient management strategies.

## CRediT authorship contribution statement

**Amandeep Singh Josan:** Writing – original draft, Visualization, Validation, Methodology, Investigation, Formal analysis, Data curation, Conceptualization. **Laura J. Taylor:** Writing – original draft, Investigation, Data curation. **Shabnam Raji:** Writing – original draft, Data curation. **Jasmina Cehajic-Kapetanovic:** Writing – review & editing, Data curation. **Robert E. MacLaren:** Writing – review & editing, Supervision.
